# Prefrontal-amygdala fear networks come into focus

**DOI:** 10.3389/fnsys.2015.00145

**Published:** 2015-10-30

**Authors:** Maithe Arruda-Carvalho, Roger L. Clem

**Affiliations:** Fishberg Department of Neuroscience and The Friedman Brain Institute, Icahn School of Medicine at Mount SinaiNew York, NY, USA

**Keywords:** intercalated, central amygdala, paraventricular thalamus, basal amygdala, prelimbic, infralimbic, consolidation, optogenetics

## Abstract

The ability to form associations between aversive threats and their predictors is fundamental to survival. However, fear and anxiety in excess are detrimental and are a hallmark of psychiatric diseases such as post-traumatic stress disorder (PTSD). PTSD symptomatology includes persistent and intrusive thoughts of an experienced trauma, suggesting an inability to downregulate fear when a corresponding threat has subsided. Convergent evidence from human and rodent studies supports a role for the medial prefrontal cortex (mPFC)-amygdala network in both PTSD and the regulation of fear memory expression. In particular, current models stipulate that the prelimbic (PL) and infralimbic (IL) subdivisions of the rodent mPFC bidirectionally regulate fear expression via differential recruitment of amygdala neuronal subpopulations. However, an array of recent studies that employ new technical approaches has fundamentally challenged this interpretation. Here we explore how a new emphasis on the contribution of inhibitory neuronal populations, subcortical structures and the passage of time is reshaping our understanding of mPFC-amygdala circuits and their control over fear.

## Introduction

Exposure to threats triggers a powerful form of learning that mediates the expression of survival-promoting behavior. The neural circuitry underlying this learning has been dissected largely through the use of Pavlovian fear conditioning, in which animals develop an association between a neutral conditioned stimulus (CS) and an aversive unconditioned stimulus (US; Maren, 2001). After training, CS presentations evoke arousal as well as a range of quantifiable defensive behaviors such as freezing (Blanchard and Blanchard, 1969). However, continued CS exposure leads to extinction, reflecting a neural process for attenuating fear when a corresponding threat has subsided. Evidence suggests that bidirectional regulation of conditioned fear involves both the amygdala and medial prefrontal cortex (mPFC), densely interconnected structures where abnormal activity has been associated with exaggerated, overgeneralized, and extinction-resistant emotional responses in conditions like post-traumatic stress disorder (PTSD; Shin et al., 2006). Rodent-based studies into the anatomy, neurophysiology and plasticity of mPFC-amygdala networks may thus lay the groundwork for alleviating maladaptive fear by correcting dysfunctional emotional learning.

## Conventional View of mPFC-Amygdala Network Function

A primary point of integration for auditory, somatosensory and nociceptive information in the amygdala is the basolateral complex (BLA; comprised of the lateral, basal, and basomedial nuclei), which plays a central role in fear responding to discrete cues as well as contexts. BLA cell types include principal excitatory neurons and a sparse, heterogeneous population of GABAergic interneurons (McDonald, 1982). Principal neurons provide direct and indirect output to the central nucleus (CeA; Amano et al., 2010), a major relay to brain systems responsible for motor and autonomic aspects of fear. Extensive and reciprocal projections exist between BLA and mPFC (Little and Carter, 2013), which in rodents is comprised of the prelimbic (PL), infralimbic (IL), anterior cingulate (ACC) and medial agranular cortices (Ongur and Price, 2000). Importantly, there is comparatively little evidence of mPFC connectivity with CeA (McDonald et al., 1996), suggesting that mPFC regulates fear expression largely through neural integration in the BLA.

Despite early indications of its involvement in fear processing, definitive evidence linking mPFC to fear conditioning has begun to emerge only recently (Courtin et al., 2013). Subsequent anatomically selective manipulations have clarified a divergence within prefrontal subregions in the control of fear expression. Extinction is impaired by IL lesion and pharmacological inactivation (Quirk et al., 2000; Chang and Maren, 2010; Fontanez-Nuin et al., 2011; Sierra-Mercado et al., 2011; Santini et al., 2012), but potentiated by IL stimulation (Milad et al., 2004; Vidal-Gonzalez et al., 2006; Kim et al., 2010; Maroun et al., 2012). Accordingly, IL unit activity positively correlates with fear inhibition (Milad and Quirk, 2002; Barrett et al., 2003; Burgos-Robles et al., 2007). In contrast, PL firing tracks CS-evoked freezing (Burgos-Robles et al., 2009; Sotres-Bayon et al., 2012; Courtin et al., 2014), which is reduced or enhanced by PL lesion (Corcoran and Quirk, 2007; Sierra-Mercado et al., 2011) or stimulation (Vidal-Gonzalez et al., 2006), respectively.

**Figure 1:**
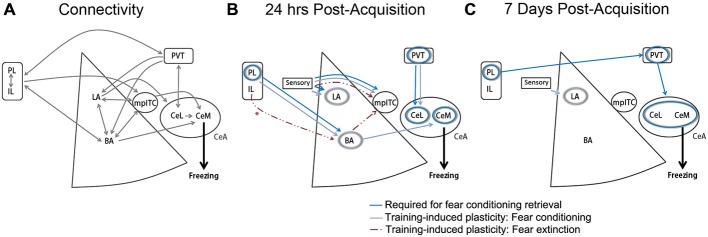
**Medial prefrontal cortex (mPFC)-amygdala contributions to conditioned fear and extinction. (A)** Established monosynaptic connections between mPFC and amygdala subregions. Double arrows indicate bidirectional projections. **(B)** Model depicting brain regions (circles) or projection pathways (arrows) implicated in fear conditioning or extinction, as expressed at 24 h after fear or extinction training. *IL-BLA projections display plasticity at 24 h after extinction, and have been shown to be necessary for extinction acquisition, but are not required for extinction retrieval at 24 h after acquisition (Cho et al., 2013; Do-Monte et al., 2015a). **(C)** Model depicting brain regions (circles) or projection pathways (arrows) implicated in fear conditioning, as expressed at 7 days after fear conditioning. Designation of regions and pathways required for retrieval is based on the results of *in vivo* cell- or projection-specific optogenetic or chemogenetic manipulations. Designation of pathways displaying training-associated synaptic plasticity is based on *ex vivo* brain slice recordings obtained at 24 h or 7 days after fear conditioning or extinction. BA, basal amygdala; CeA, central amygdala; CeL, lateral division of the central amygdala; CeM, medial division of the central amygdala; IL, infralimbic cortex; LA, lateral amygdala; mpITC, medial paracapsular intercalated cells; PL, prelimbic cortex; PVT, paraventricular thalamus. Additional references used to compile these models, but not discussed within the main text, include (Amano et al., 2010; Clem and Huganir, 2010; Nabavi et al., 2014).

Current models attribute this dichotomy in IL and PL function to their differential recruitment of downstream BLA targets. In particular, a central role in fear inhibition has been ascribed to the intercalated cell masses (ITCs; Berretta et al., 2005; Marek et al., 2013), a cluster of inhibitory cells in BLA that project to CeA (Millhouse, 1986; Sah et al., 2003). Anterograde tracing studies have suggested that while both PL and IL project to the amygdala, IL axons arborize more extensively in the vicinity of ITCs (McDonald et al., 1996; Pinard et al., 2012), in which electrophysiological recordings have also revealed neural responses to IL stimulation (Amir et al., 2011). Coupled with the observation that neurotoxic ITC ablation impairs extinction (Likhtik et al., 2008), these studies led to the interpretation that IL forms an extinction circuit with ITCs. Enabled by optogenetic and chemogenetic advances, a wave of recent reports have effectively supplanted this model and suggested a system that is considerably more complex (Figure 1).

## New Concepts Emerge

Contrary to previous anatomical tracing, several new studies appear to reveal similar profiles of PL and IL axon terminals within BLA following mPFC injection of channelrhodopsin-expressing viral vectors (Cho et al., 2013; Arruda-Carvalho and Clem, 2014; Hübner et al., 2014). The most prominent target of PL and IL axons appears to be the anterior basal nucleus, where optic terminal stimulation in either pathway evokes monosynaptic excitation and feedforward inhibition in BLA principal neurons. Importantly, neither the strength of excitation nor feedforward inhibition was shown to differ between PL and IL pathways. By comparison, Cho et al. (2013) reported sparse responses to terminal stimulation in ITCs (but see Strobel et al., 2015), suggesting the basal nucleus is the principal, convergent target of PL and IL projections in the amygdala.

Casting further doubt on the IL-ITC model of extinction, the use of optogenetic electrophysiology has also helped identify new plasticity mechanisms that may underlie ITC encoding of both fear expression and inhibition. While fear conditioning and extinction do not affect mPFC synapses onto ITCs (Cho et al., 2013), both processes were shown by Asede et al. (2015) to be associated with plasticity of ITC synapses formed by afferents of thalamic and neocortical sensory regions. Given that ITC cells can drive feedforward and feedback inhibition in BLA principal neurons (Asede et al., 2015), future research should elucidate whether physiological modulation of fear expression and BLA activity is achieved through thalamic or cortical engagement of ITC cells and at what stages of fear memory processing this might occur.

Within principal neurons of the basal nucleus, evidence indicates that plasticity of mPFC synapses contributes to bidirectional fear regulation. A study from our group demonstrated that fear encoding leads to selective postsynaptic strengthening in the PL-BLA pathway (Arruda-Carvalho and Clem, 2014). Conversely, Cho et al. (2013) also showed that extinction leads to a decrease in glutamate release probability of mPFC-BLA synapses. Although injections in this study were targeted to IL, viral leakage into PL precluded localization of this plasticity to a specific pathway. Together, however, these studies outline a role for mPFC-BLA plasticity in encoding of fear and extinction, but it remains unclear precisely how this plasticity alters neural integration by BLA projection neurons to support fear expression or inhibition. The description within BLA of intermingled fear and extinction-related neurons (Herry et al., 2008), which exhibit differences in CS-evoked firing as well as downstream projection targets (Senn et al., 2014), suggests the possibility of a hardwired substrate for cell-selective plasticity within PL and IL pathways.

Complicating the situation, however, two intriguing studies from Do-Monte and colleagues suggest that long-term fear expression and inhibition may not be mediated by mPFC-BLA networks. According to the first study, while PL activity mediates retrieval of conditioned fear, the downstream region engaged by PL neurons changes over time (Do-Monte et al., 2015b). Optogenetic silencing of PL-BLA projections interferes with fear expression at 6 h, but not at 7 days after fear conditioning. Paralleling this loss of PL-BLA mediation, fear retrieval instead becomes dependent on PL transmission in the paraventricular thalamus (PVT). Such a temporal shift in PL synaptic processes calls into question the relevance of PL-BLA circuits to persistent fear memories that characterize PTSD, and points to a potential role for systems-level consolidation in the development of that pathology. Interestingly, in a separate study the same group showed that optogenetic somatic inhibition of IL neurons modulates extinction only when applied at training, but not at retrieval of extinction one day or one week later (Do-Monte et al., 2015a). A subsequent report in which optogenetic manipulation was targeted more specifically to IL terminals in the BLA confirmed a role for this pathway in acquisition but not retrieval of extinction (Bukalo et al., 2015). This would seemingly represent a complete refutation of IL-BLA involvement more generally, or IL-ITC circuits specifically, in mediating the expression of long-term extinction memory. Indeed, both studies by Do-Monte and colleagues are consistent with the notion that mPFC-BLA pathways may function mainly to orchestrate emotional memory storage by other brain circuits. Dissection of the time-dependent contributions of other pathways to fear expression and inhibition could help clarify the precise role of mPFC subregions in guiding these processes.

The paraventricular region of the dorsal midline thalamus (PVT) has emerged as a potential key player in long-term memory expression with reciprocal connectivity to the CeA (Penzo et al., 2014, 2015), a structure long considered indispensable for conditioned fear responses. PVT neurons start to show CS responsivity at around 24 h after fear conditioning (Do-Monte et al., 2015b). Correspondingly, pharmacological inactivation of PVT impairs fear retrieval at this time point (Padilla-Coreano et al., 2012; Do-Monte et al., 2015b), but does not interfere with extinction (Padilla-Coreano et al., 2012). In addition, projection-specific optogenetic and chemogenetic manipulations show that both afferent and efferent connections in PVT are required for fear expression in the intermediate to remote term. Specifically, these studies indicate that fear retrieval requires PL-PVT inputs at 7 days, as well as PVT-CeA outputs at both 24 h and 7 days (Do-Monte et al., 2015b; Penzo et al., 2015). With regard to the latter pathway, PVT neurons were also shown to preferentially synapse onto somatostatin-expressing (SOM+) lateral division of the central amygdala (CeL) interneurons (Penzo et al., 2015), a population previously shown to mediate fear expression (Li et al., 2013). Presumably, GABA released by SOM+ interneurons ultimately disinhibits medial division of the central amygdala (CeM) neurons to gate fear responses.

While these data paint a picture in which PVT-CeA circuits come into play only after a period of initial memory consolidation, electrophysiological recordings suggest their involvement very early in fear encoding. Penzo and colleagues showed that activity of CeA-projecting PVT neurons releases BDNF in the CeA and is required for enhancement of excitatory input onto SOM+ CeL neurons, which manifests as early as 3 h after fear conditioning (Li et al., 2013; Penzo et al., 2015). Furthermore, pretraining inhibition of CeA-projecting PVT cells impairs auditory fear conditioning (Penzo et al., 2015). These data suggest that while it may be more heavily engaged following fear memory consolidation, PVT-CeL signaling also mediates early plasticity required for subsequent fear memory retrieval. Given that PVT projects to PL, IL, ventral hippocampus (vHPC) and BLA (but very weakly to LA) (Li and Kirouac, 2008; Vertes and Hoover, 2008), future work clarifying how (and when) this structure converses with regions other than PL and CeA should help determine the overall contribution of PVT in modulating fear conditioning and extinction.

## Conclusion

On the one hand, the above studies create new ambiguity about the precise temporal contributions and downstream targets of PL and IL neurons in fear regulation, but they also conceptually advance our understanding of fear regulation by introducing new players to the dance. A future challenge will be to elucidate the complex choreography of different projection populations (IL, PL, BLA, ITC, CeA, PVT, and vHPC, among others) that ultimately gives rise to long-term fear and extinction memory. This will in turn help pinpoint with better clarity the dysfunctional mechanisms of fear processing that underlie conditions like PTSD. The new insights described here suggest that while these disorders may involve some degree of mPFC and BLA dysfunction, they might ultimately be more effectively addressed by treatments directed at extended fear regulatory circuits that are nevertheless heavily influenced by mPFC-BLA networks.

## Funding

This work was supported by NIH grant MH105414 (RLC), a Young Investigator Award from the Brain and Behavior Research Foundation (RLC), and a Human Frontiers Science Program Long-Term Fellowship (LT000191/2014-L) (MAC).

## Conflict of Interest Statement

The authors declare that the research was conducted in the absence of any commercial or financial relationships that could be construed as a potential conflict of interest.
